# A systematic review and activation likelihood estimation meta-analysis of fMRI studies on arousing or wake-promoting effects in Buddhist meditation

**DOI:** 10.3389/fpsyg.2023.1136983

**Published:** 2023-10-27

**Authors:** Inder S. Chaudhary, Gary Chon-Wen Shyi, Shih-Tseng Tina Huang

**Affiliations:** ^1^PhD Program in Cognitive Sciences, National Chung Cheng University, Chiayi City, Taiwan; ^2^Center for Research in Cognitive Sciences, National Chung Cheng University, Chiayi City, Taiwan; ^3^Department of Psychology, National Chung Cheng University, Chiayi City, Taiwan

**Keywords:** alertness, wakefulness, arousing, fMRI, meta-analysis, Buddhist meditation

## Abstract

Conventional Buddhist texts illustrate meditation as a condition of relaxed alertness that must fend against extreme hypoarousal (sleep, drowsiness) and extreme hyperarousal (restlessness). Theoretical, neurophysiological, and neuroimaging investigations of meditation have highlighted the relaxing effects and hypoarousing without emphasizing the alertness-promoting effects. Here we performed a systematic review supported by an activation-likelihood estimate (ALE) meta-analysis in an effort to counterbalance the surfeit of scholarship emphasizing the hypoarousing and relaxing effects of different forms of Buddhist meditation. Specifically, the current systematic review-cum-meta-analytical review seeks to highlight more support for meditation’s wake-promoting effects by drawing from neuroimaging research during wakefulness and meditation. In this systematic review and meta-analysis of 22 fMRI studies, we aim to highlight support for Buddhist meditation’s wake-promoting or arousing effects by identifying brain regions associated with alertness during meditation. The most significant peaks were localized medial frontal gyrus (MFG) and precuneus. We failed to determine areas ostensibly common to alertness-related meditation such as the medial prefrontal cortex (mPFC), superior parietal lobule, basal ganglia, thalamus, most likely due to the relatively fewer fMRI investigations that used wakefulness-promoting meditation techniques. Also, we argue that forthcoming research on meditation, related to alertness or wakefulness, continues to adopt a multi-modal method to investigate the correlation between actual behaviors and neural networks connected to Buddhist meditation. Moreover, we recommend the implementation of fMRI paradigms on Buddhist meditation with clinically diagnosed participants to complement recent trends in psychotherapy such as mindfulness-based cognitive therapy (MBCT).

## Introduction

### Meta-analytical evidence of arousing or wake-promoting effects in meditation

Meditation can be defined as a set of mental workouts-cum-exercises that regulate cognition, the self, and emotion, where a specific directing of attention and awareness influences mental and related somatic events. The practice of meditation has gained significant attention in recent years due to its potential to regulate cognition, emotion, and the self. Derived from Buddhist traditions, meditation involves specific directing of attention and awareness toward mental and somatic events, leading to a state of mind characterized by reduced negative emotions and improved positive states of mind (Goleman, 1977; Cardoso et al., 2004). Research in neuroscientific and behavioral investigations has shown the significance of studying meditation states and traits to acquire improved learning of attention, affective and cognitive neuroplasticity, awareness, and various states of mind (Adolphs, 2003; Cahn and Polich, 2006; Lutz et al., 2008a; Raffone and Srinivasan, 2010; Montero-Marin et al., 2019; Raffone et al., 2019). As such, clinical applications of meditation-based mental training initiatives are increasingly being devised and evaluated in various medical contexts and conditions (Kabat-Zinn, 1982; Cahn and Polich, 2006; Hofmann et al., 2010; Lin et al., 2018; Tang and Tang, 2020).

The popularity of meditation programs and practices derived from Buddhist traditions in the West has resulted in these practices being primarily deconstructed from their actual Buddhist meditational goal of “awakening” (Brown and Ryan, 2003; Lopez, 2009; Britton et al., 2014; Kabat-Zinn, 2018) and reconstructed as clinical interventions or forms of “psychological and physiological self-maintenance” (Beary and Benson, 1974; McMahan, 2008; Kabat-Zinn, 2013; Raffone et al., 2019; Tang and Tang, 2020). This has brought about some alterations in the traditional goals of the meditation practices and impacted how scientific work documents the effects of Buddhist meditation (Davidson, 2010; Williams and Kabat-Zinn, 2011; Kabat-Zinn, 2013; Lin et al., 2018; Tang and Tang, 2020). For instance, several Tibetan Buddhist scriptures explain the act of meditation as cultivating a state of mind of relaxed alertness (Amihai and Kozhevnikov, 2014). This mental state is about maintaining a continuous balance between hypoarousal, sleep, excessive relaxation, and mental dullness on one end and hyperarousal, agitation, and restlessness on another (Pandita, 1992; Gunaratana, 2002; Kozhevnikov et al., 2009; Bodhi, 2012; Amihai and Kozhevnikov, 2015; Kozhevnikov, 2019). Moreover, the focus has been given more to the relaxation effects of Buddhist meditation within the modern context without as much attention given to the wake-promoting or arousing effects. Thus, it is the aim of this study to highlight support for the wake-promoting or arousing effects of Buddhist meditation by identifying brain regions associated with alertness during meditation. This is achieved by performing an interdisciplinary systematic cum meta-analytical review to highlight evidence for the wake-promoting or arousing effect of Buddhist meditation.

It is observed that in some recent research, the objective of meditation work has aimed beyond relaxation and decreased arousal (Kozhevnikov et al., 2009; Amihai and Kozhevnikov, 2015; Lin et al., 2018; Basso et al., 2019; Chaudhary, 2019; Kozhevnikov, 2019). Such research includes a consciousness study (Williams and West, 1975; Fenwick et al., 1977; Cahn and Polich, 2006), a sleep equivalence study (Pagano et al., 1976), or a type of hibernation study (Younger et al., 1975) that is flipped by wakefulness (Walker and Berger, 1980). These types of studies usually associate meditation practices with non-specific relaxation techniques (Cooper and Aygen, 1979), where sleep and hypoarousal are seen as a fit instead of an impediment to concentration (Kyabgon, 2003).

When it comes to hypoarousal-related activities, Buddhism views attentive wakefulness as a significantly deep shift in a practitioner’s insight. This shift in perception is called “awakening.” The balance between mental laxity and agitation is achieved through meditation. The act of meditation helps the practitioner to reach a relaxed state that is imbued with zeal, courageous energy, and an alert and sharp awareness in a manner that the meditator is committed to wakefulness and fended against the fetters of sleepiness and tiredness (Nanamoli and Bodhi, 1995; Gyatso, 2001). Buddhist scholars, monks, and enthusiasts acquainted with conventional Buddhist objectives of meditation have criticized Western works on Buddhist meditation practice for their exaggeration of the relaxation part of meditation, which substantially overshadowed other aspects involved in Buddhist meditation (Wallace, 2006; McMahan, 2008).

While meditation programs and practices have gained popularity in the West, their original goals from Buddhist meditation have been altered to fit contemporary needs. However, some modern-day research aims to explore beyond relaxation and decreased arousal associated with meditation practices. Future studies should aim to identify brain regions associated with the wake-promoting or arousing effects of Buddhist meditation, in addition to exploring traditional Buddhist goals, to comprehensively understand the effects of meditation on cognitive (Chaudhary, 2018), affective, and physiological functions. Further, the present study seeks to address the gap in the literature by examining the wake-promoting effects of Buddhist meditation, a less explored aspect of meditation practice. The identification of brain regions associated with alertness during meditation could provide important insights into the mechanisms underlying the wake-promoting effects of meditation, potentially leading to the development of targeted meditation-based interventions for conditions characterized by excessive daytime sleepiness or fatigue. Furthermore, this study contributes to the growing literature on the cognitive and affective benefits of meditation, which has important implications for designing clinical and educational programs to promote mental and emotional well-being.

### Association between different forms of Buddhist meditation and attention

Meditation is a widely practiced technique for improving cognitive control and emotional regulation. The different types of meditation practices are often classified into two styles: Focused Attention Meditation (FAM) and Open Monitoring Meditation (OMM). FAM involves focused attention on a given object, while OMM emphasizes open monitoring of experiences (Fujino et al., 2018; Raffone et al., 2019; Engström et al., 2022). In recent years, Loving-Kindness Meditation (LKM) and Compassion Meditation (CM) have gained importance as a third type of meditation, with an emphasis on specific intentions and sentiments (Kakumanu et al., 2019; Raffone et al., 2019; Engström et al., 2022).

Focused Attention Meditation involves sustained attention to a given object. This type of meditation is concerned with achieving a mental state of concentrated, serene focused attention, called “*Samatha*” (in English, it means quiescence) in Buddhist meditative practice. FAM also involves modulating attentional focus and noticing distractions generated by internal or external sources (Brandmeyer et al., 2019; Raffone et al., 2019; Engström et al., 2022). The monitoring and attentional processes of FAM have been associated with various neural networks involved in sustained and selective attention and attentional control (MacLean et al., 2010). A functional magnetic resonance imaging (fMRI) study conducted by Hasenkamp et al. (2012) revealed significant neural correlations of FA functions. This fMRI investigation also pinpointed distinct brain areas and systems associated with awareness, mind wandering, and shifting attention to and from the object.

Open Monitoring Meditation (OMM) involves non-reactive observation of experiences. This type of meditation is concerned with witnessing upcoming feelings, thoughts, and sensory occurrences within an open “background” of attention. In the event of shifting from an FAM to an OMM form of meditation, the item as the main attention is slowly substituted by maintaining an open presence or awareness (Lutz et al., 2007, 2008a). Behavioral research has revealed that OMM practitioners have improved conflict monitoring and more distributed attentional focus (Chan and Woollacott, 2007; Slagter et al., 2007; Tang et al., 2007; Tang and Tang, 2020). And they exhibit a reduced attentional blink or a more efficient way of allocating resources to serially presented positions. Further, OMM can lead to regulatory impacts on emotional functions from prefrontal modulation of limbic reactions (Hariri et al., 2000; Lieberman et al., 2007; Tang and Tang, 2020 see pp. 109–132).

Apart from FA and OM styles of meditation, LKM and CM are the other two notable types of Buddhist meditation. The practice involves unconditionally executing the sensations and concerns to oneself, other distinctive beings, or all living beings. Visualizing the mental image of the objects or intrinsic rays of light from one’s heart to others or concentrically everywhere to induce compassion and loving-kindness sensations and intentions is an essential component of LKM and CM practices (Khantipalo, 1984; Sujiva, 1991; Raffone et al., 2019). These types of meditation are usually practiced in fitting coordination with OM and FA to decrease unwholesome mental states (e.g., hatred, anger) and develop virtuousness (Buddharakkhita, 1995; The Dalai Lama, 2001; Hofmann et al., 2011).

Expert practitioners of LKM or CM trigger limbic regions and brain systems linked with processing mental states of other individuals (medial PFC, temporal lobes, posterior cingulate cortex, and temporoparietal junction), especially in the right hemisphere. Highly intense forms of CM activate the right inferior parietal lobule in the medial-posterior part of the right insula, dorsal ACC, and the somatosensory cortices (Lutz et al., 2009; Tang and Tang, 2020). Similarly, novice meditators given lessons in a mindfulness-based stress reduction (MBSR) program showed activation in the inferior parietal lobule and viscero-somatic areas such as the insula, right-lateralized network involving the lateral PFC and secondary somatosensory cortex when asked to perform experiential focusing (present moment awareness) (Kabat-Zinn, 1990, 2013; Farb et al., 2007; Baer, 2013).

Furthermore, bilateral activation in the dorsal medial prefrontal cortex and the rostral anterior cingulate cortex was noticed in Vipassana experts compared to beginners (Hölzel et al., 2007). The act of focusing on breathing during the auditory task deactivated the default mode network (DMN) (Buckner et al., 2008) in experienced Zen practitioners in comparison to beginners (Pagnoni et al., 2008). DMN is a set of neural networks that are operative when one is not involved in a work-induced exercise. It has been suggested that DMN is involved in self-referential functions (Buckner and Vincent, 2007; Qin and Northoff, 2011; Shi and He, 2020).

During breath-focused meditation (a form of FA style of meditation) (Pozuelos et al., 2019), it is observed that there is an increase in the BOLD signal in the right and left putamen and the supplementary motor area, and a decrease in activation in the right precuneus, the parietal-temporal cortex, and posterior cingulated cortex. Sustained meditation (falls under the umbrella term of FA type of meditation) increases neural activity in the head of the caudate nucleus bilaterally (Baerentsen, 2001; Baerentsen et al., 2010). In mindfulness meditation (a subjective form of meditation - may or may not include one or more types of meditation) (Grossman, 2010; Chiesa, 2012), it is also observed to decrease neural activity in cortical midline structures (CMS) connected with interoception (bilateral precuneus, right medial prefrontal cortex, and bilateral anterior insula) and substantially increase activity in the right posterior cingulate cortex (Ives-Deliperi et al., 2011).

In other meditation studies, researchers examined Theravada Buddhist monks, who were experienced in both Vipassana (OM/LKM/CM) and Samatha (FA) meditations and noticed that Samatha induces a series of increased activations in the right medial anterior prefrontal cortex and the right and left anterior cingulate cortex while witnessing deactivations in the left hemisphere (Manna et al., 2010). In contrast, those practicing only Vipassana meditation demonstrated activations in the left hemisphere, including the superior temporal gyrus, medial anterior prefrontal cortex, and superior parietal lobule. Similarly, expert Zen practitioners who mindfully observed emotional pictures exhibited a deactivation pattern in the default mode network, unrelated to brain areas responsible for emotional activity (Taylor et al., 2011). Other studies have shown that awareness of mind wandering activates the dorsal anterior cingulate cortex and bilateral anterior insula, while shifting attention from mind wandering to breathing activates the lateral inferior parietal cortex and the lateral and dorsal prefrontal cortex, with stronger activations in the right hemisphere. Sustaining focused attention on breathing activates the dorsolateral prefrontal cortex, which is associated with executive attention (Hasenkamp et al., 2012).

With reference to well-established approaches to the study of attention, attention can be divided into conflict monitoring or executive attention, alerting, and orienting attention (Posner, 2017). Alerting affects the noradrenaline brain network while orienting attention involves the brain’s parietal and frontal areas, including the frontal eye fields and inferior and superior parietal lobes. Conflict monitoring and resolution involve the anterior insula (AI), anterior cingulate cortex (ACC), and basal ganglia (Raz and Buhle, 2006; Braboszcz and Delorme, 2011; Petersen and Posner, 2012; Malinowski, 2013; Tang and Tang, 2020).

In this study, we discuss the phenomenon of alerting, which refers to achieving and sustaining a state of mental readiness that is highly responsive to incoming stimuli. Alerting can be categorized into two types: phasic alertness, which involves enhancing reaction following a warning signal or cue, and tonic alertness, also known as vigilant attention or intrinsic alertness (Langner and Eickhoff, 2013), which refers to the average level of vigilance, arousal, wakefulness, alertness, or mental state of readiness to notice or react to irregular or unanticipated cues. Tonic alertness serves as the cognitive tone for carrying out more complex processes, such as executive control and working memory, and is a fundamental requirement for other forms of attention (Sturm and Willmes, 2001). Previous research has shown that meditation can improve different types of attention, but less work has been done on tonic alertness. While Western scholarship tends to focus on the relaxation benefits of meditation, the positive correlation between arousal/wakefulness and attention has been well-supported in sleep and chronobiology research (Killgore, 2010). The relationship between arousal and meditation is a relatively new area of study in the field.

### The link between attention and wakefulness

In the field of meditation, it is mainly observed that the deliberate training of attention (either in an open and receptive or focused and directed way) is an essential part of any meditation. This systematic review underlines open monitoring (OM) and focused attention (FA), the two common forms of attentional training that are paramount in Buddhist and many non-Buddhist meditational practices (Lutz et al., 2008b,^2014^; Guendelman et al., 2017; Sumantry and Stewart, 2021).

Tonic alertness (Langner and Eickhoff, 2013) is a broad level of alertness, arousal, wakefulness, vigilance or mental state of preparedness to respond to/notice unexpected or irregular stimuli (Fan et al., 2003; Oken et al., 2006). Another way to measure tonic alertness is by its deficit, which is a basic level of sleepiness, absence of sustained attention, or weariness depicted by mind wandering (Stawarczyk et al., 2011) or an attentional lapse (Sturm and Willmes, 2001; Fan et al., 2002; Weissman et al., 2006; Clemens et al., 2011; Arvidsson, 2018). This measure is possible as tonic alertness delivers the cognitive tone for serving more complicated functions, such as executive control and working memory (Posner, 2008; Clemens et al., 2011). Further, all the above-mentioned attention forms depend on tonic alertness (Raz and Buhle, 2006). Moreover, it is worth noticing that research in the pharmacological enhancement of alertness (Valentine and Sweet, 1999; Arnsten and Pliszka, 2011; Pinggal et al., 2022) or alertness training (Sturm et al., 2006; Melnychuk et al., 2018) showing improvements on a vast number of attentional tasks also supports the idea of assuming tonic alertness as a foundational prerequisite for other forms of attention. There exist a plethora of work done on all three types of attention (i.e., executive attention, alerting, and orienting attention) that have been researched in meditation studies (Chambers et al., 2008; Bushell, 2009; Lutz et al., 2009; Prakash et al., 2020; Tanaka et al., 2021), less emphasis has been placed on tonic alertness (Kozhevnikov et al., 2022).

Likewise, tonic alertness has been expressed in terms of improved vigilance or sustained attention (Jha et al., 2007; Kaul et al., 2010; Kozhevnikov, 2019; Valdez, 2019; Simor et al., 2020; Jaeger et al., 2021) but not in terms of increased arousal or wakefulness, an approach influenced by the modern western view that meditation stimulates sleep and relaxation rather than wakefulness or arousal. The favorable association between attention and arousal or wakefulness is apparent in everyday affairs. We all have witnessed, for example, that paying attention, learning, recalling, and regulating emotions are more challenging when we are exhausted or that extremely engaging in attentive activities before going to bed can hinder one’s sleep. The notion that attention is positively correlated with greater arousal and wakefulness is well-established within the fields of sleep and chronobiology (Killgore, 2010; Whitney et al., 2019; Hudson et al., 2020; Mishra et al., 2020), but this correlation has been somewhat less investigated within the domain of meditation research. Thus, this systematic review will explore the effects of Buddhist meditation on the index of sleep proneness or wakefulness, i.e., the activation of wake or sleep-corresponding brain regions.

Our study presents an activation-likelihood estimate (ALE) meta-analysis of functional neuroimaging studies on alertness-related meditation. Our study aims to integrate prior fMRI works on meditation that induce arousal or wakefulness effect, yielding insight into the brain networks that are most commonly associated with alertness-related meditation techniques. To identify studies for the meta-analysis, we included 22 fMRI studies that analyzed activation foci reflecting arousal, wakefulness, or alertness-related activation. We analyzed the operationalization of meditation techniques and experimental paradigms employed by these studies, allowing us to identify disparities within the brain networks associated with various aspects of alertness-related meditation.

## Materials and methods

### Search strategy (literature search and data sources)

**FIGURE 1:**
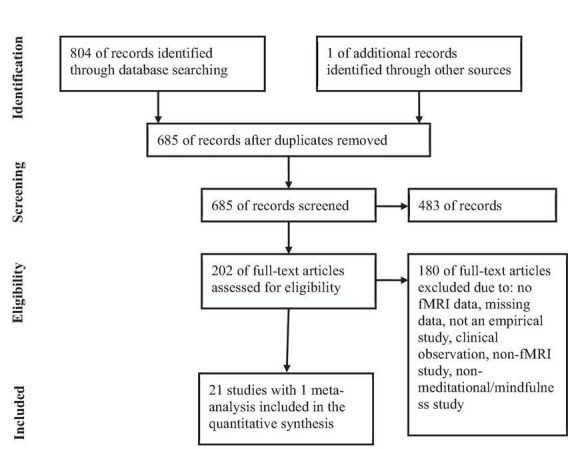
fMRI studies were screened and selected as per the PRISMA flow chart. Selection of fMRI studies was carried out in accordance with PRISMA guidelines for reporting systematic reviews (Moher et al., 2009).

PUBMED database was searched for functional imaging studies focusing on effects of Buddhist Meditation (BM) and Buddhist-derived Meditation Techniques (BdMT) on brain regions underlying tonic alertness or relaxation. As of October 2, 2021, the search terms were the following: ((“buddhis*” [all fields] or “buddhism” [all fields]) + (“meditation” [all fields]) + (“fMRI” [all fields] or “fMRI” [MeSH])); ((“buddhis*” [all fields] or “buddhism” [all fields]) + (“meditation” [all fields]) + (“neuroimaging” [all fields] or “neuroimaging” [MeSH])); ((“buddhis*” [all fields] or “buddhism” [all fields]) + (“meditation” [all fields]) + (“PET” [all fields] or “PET” [MeSH])); ((“buddhis*” [all fields] or “buddhism” [all fields]) + (“meditation” [all fields]) + (“neuro*” [all fields] or “neuro*” [MeSH])) and ((“mindfulness” [all fields] or “mindfulness” [MeSH]) + (“fMRI” [all fields] or “fMRI” [MeSH])). Searches were without limit on publication date and language. Also, the Boolean operators “OR,” and + was used to make the search more precise and accurate. Using of ‘AND’ was avoided because the search engine use this particular Boolean operator by default. The asterisk ‘*’ was used as a wildcard character to search for all words that contain the letters entered. For example, neuro* would find “neuroimaging” or “neurology” or “neuropathy.” A total of 685 exclusive fMRI studies were acknowledged (see Figure 1).

### Study selection/eligibility screening/eligibility criteria

The following are the inclusion-exclusion criteria for the systematic review-cum-meta-analyses:

(1) Articles that cited relaxation, arousal, tonic alertness, alertness, Buddhism, neuroimaging, mindfulness, and fMRI within the keywords, abstract, and title were considered and articles that did were rejected. At this screening stage, 202 of the 685 articles met the criterion.
(2) Research articles that employed fMRI were considered, whereas the work that utilized other techniques (i.e., EEG, PET, SPECT, TMS, behavioral efforts, and review articles) was rejected. Additionally, references of screened articles were carefully searched to identify more studies dealing with the brain systems responsible for arousal in Buddhist meditation practices. Further, the inclusion criteria held clinical sound adults and experiments where participants performed BM/BdMT during the fMRI procedure. Amidst a total of 202 studies, only 33 papers met this criterion.
(3) In the final screening process, only studies reporting whole-brain group analyses in a common reference space were considered. It was checked that the study performed a random-effects analysis. Further, both studies addressing only ROI analysis and no coordinates (*N* = 6), and anatomical studies showing structural changes (*N* = 7) were removed. This exercise ensured that the chosen neuro-imaging contrasts were as similar as possible (e.g., meditation vs. rest). However, when this homogeneity was not feasible, other comparisons were considered in the selecting articles (like meditation vs. control task). Following the above-stated criteria, data from a total of 22 papers (see Table 1 for the listing of all included studies) were included in the meta-analytical study.

**TABLE 1:** Meditation studies depicting the effects of meditation practice on the brain regions related to alertness.

SN	CN	Meditation studies	Meditation type	Study design	Task	Sample	Scanner	Contrast	Foci	DLPFC	dACC	AIC	IPL	SPL	BG	THAL
1	1	Allen et al., 2012	6-week Mindfulness	LRCT, CS	Affective Stroop at R	NOV	3T	M vs Active-Control	18 A	↑L	↑	↑R				
2	2	Baerentsen et al., 2010	Zen, Yoga, Tibetan	CSwC	On-Off Meditation	EX	1.5T	Onset: M vs. R	13 A; 12 D				↑B	↓R	↑B	
	3	Baerentsen et al., 2010	Yoga, Tibetan	CSwC	Continuous Meditation	EX	1.5T	Sustained: M vs. R	1 A; 33 D	↓R					↑	
3	4	Bauer et al., 2019	Vipassana	CS	M	EX, NOV	3T	Meditation trait effects: EM rsBase > HC rsBase	6 D	↓B						
	5	Bauer et al., 2019	Vipassana	CS	M	EX, NOV	3T	Meditation state effects: EM Med > EM rsBase	2 A	↑B						
	6	Bauer et al., 2019	Vipassana	CS	M	EX, NOV	3T	State-to-trait effects: EM rsPost > EM rsBase	2 A	↑B						
4	7	Brewer et al., 2011	FA, OM, LKM	WS, CS	M	EX, NM	3T	M vs. R	11 D					↓		
5	8	Dickenson et al., 2013	Mindfulness	CSwC	MM	NOV	3T	FB > MW correlated with MAAS	3 A	↑R			↑R	↑R		
6	9	Doll et al., 2016	Attention to Breathing	CS	EET	NOV	3T	Attention-To-Breath > Passive Viewing	7 A	↑L	↑L					
	10	Doll et al., 2016	Attention to Breathing	CS	EET	NOV	3T	(Attention-To-Breath > Passive Viewing) picture	20 A		↑B		↑L			
7	11	Farb et al., 2007	8-week MBSR	CSwC	Self-focus Task	NM, NOV	3T	M while reading self-related vs. control words	4 A; 3 D	↑L		↑R				
8	12	Gard et al., 2012	Vipassana	CS	Simulation	EX	1.5T	Mf Practitioners during baseline	12 A	↓R						
	13	Gard et al., 2012	Vipassana	CS	Simulation	EX	1.5T	Receiving Electric Stimulation: Mf Practitioners > Controls	1 A		↑R					
	14	Gard et al., 2012	Vipassana	CS	Simulation	EX	1.5T	Receiving Electric Stimulation: Controls > Mf Practitioners	12 A	↑R		↑R				
	15	Gard et al., 2012	Vipassana	CS	Simulation	EX	1.5T	Anticipation of Shock: Mindfulness Practitioners > Controls	3 A	↑B	↑R					
	16	Gard et al., 2012	Vipassana	CS	Simulation	EX	1.5T	Anticipation of Shock: Controls > Mindfulness Practitioners	7 A					↓L		
9	17	Grant et al., 2011	Zen	CS	Pain-Warm Simulation	EX, NM	3T	Meditators > Controls	9 A	↓B	↑B	↑B			↑B	↑B
	18	Grant et al., 2011	Zen	CS	Pain-Warm Simulation	EX, NM		Controls > Meditators	9 A	↓B	↑B	↑B			↑B	↑B
10	19	Hasenkamp et al., 2012	FA	CSwC	M	EX	3T	AWARE vs. MW	7 A	↑L	↑B	↑B		↑L		
	20	Hasenkamp et al., 2012	FA	CSwC	M	EX	3T	SHIFT vs. MW	6 A			↓B	↑B	↓L	↑B	↑B
	21	Hasenkamp et al., 2012	FA	CSwC	M	EX	3T	FOCUS vs. MW	1 A	↑R						
11	22	Hölzel et al., 2007	Vipassana	CS	Mindfulness and Arithmetic Task	EX, NM	1.5T	Mindfulness vs. Arithmetic	6 A		↑					
12	23	Ives-Deliperi et al., 2011	MBSR	CSwC	Control Task	EX	3T	Meditation vs. Random Generation of Numbers	1 A; 7 D	↓R	↓L	↓B		↓B		
13	24	Kim et al., 2020		Meta-Analysis												
14	25	Klimecki et al., 2013	LKM (6h)	CSwC	SoVT	NOV	3T	Neutral vs. Distressing Videos at R	4 A						↑R	
15	26	Lee et al., 2012	FA, LKM	CS	CPT, EPT	EX, NOV	3T	Emotion Photos vs. Cognitive Task at R and while M	2 A	↑						
	27	Lee et al., 2012	FA	CS, WS	CPT, EPT	EX, NOV	3T	Emotion Photos vs. Cognitive Task at R and while M	2 A			↑L		↑R		↓R
	28	Lee et al., 2012	LKM	CS, WS	CPT, EPT	EX, NOV	3T	Emotion Photos vs. Cognitive Task at R and while M	2 A	↑L	↑L			↑R	↑L	
16	29	Lutz et al., 2015	Mindfulness	CS	EET	NOV, NM	3T	Mf vs. Basic (Perception negative > neutral)	2 A	↑L		↑L				
	30	Lutz et al., 2015	Mindfulness	CS	EET	NOV, NM	3T	Mf vs. Basic (Expectation negative > expectation neutral)	4 A	↑L			↑L			
	31	Lutz et al., 2015	Mindfulness	CS	EET	NOV, NM	3T	Mf vs. Basic (Expectation unknown > expectation neutral)	7 A	↑L	↑L	↑B	↑L		↑L	
17	32	Manna et al., 2010	Theravada FA	CSwC	FA-OM-R	EX, NOV	1.5T	M vs. R (Meditators FA > R)	3 A; 11 D	↑R↓L	↑B	↓L				
	33	Manna et al., 2010	Theravada OM	CSwC	FA-OM-R	EX, NOV	1.5T	M vs. R (Meditators OM > R)	3 A	↑L				↑L		
	34	Manna et al., 2010	Theravada FA, OM	CSwC	FA-OM-R	EX, NOV	1.5T	M vs. R (Meditators OM > FA)		↑B	R	↑L		↑L		
	35	Manna et al., 2010	10-Day FA	CSwC	FA-OM-R	EX, NOV	1.5T	M vs. R (NOV FA > R)								
	36	Manna et al., 2010	10-Day OM	CSwC	FA-OM-R	EX, NOV	1.5T	M vs. R (NOV OM > R)		↑R	↑B					
18	37	Opialla et al., 2015	Mindfulness	CS	EET	NOV, NM	3 T	Mf > Cognitive Control (Expectation: negative > neutral)	11 D	↑L		↑L				
	38	Opialla et al., 2015	Mindfulness	CS	EET	NOV, NM	3 T	Mf > Cognitive Control (Expectation: unknown > neutral)	1 A							
	39	Opialla et al., 2015	Mindfulness	CS	EET	NOV, NM	3 T	Mf > Cognitive Control (Perception: negative > neutral)	1 A						↑R	
19	40	Pagnoni et al., 2008	Zen	CS	M	EX, NM	3T	M vs. R	8 A							
20	41	Scheibner et al., 2017	FA	CSwC	M	NOV	3T	MW > mindful overall	4 A	↑R			↑L			
	42	Scheibner et al., 2017	FA	CSwC	M	NOV	3T	MW > mindful internal	9 A	↑R	↑			↑L		
	43	Scheibner et al., 2017	FA	CSwC	M	NOV	3T	MW > mindful external	1 A	↑R						
	44	Scheibner et al., 2017	FA	CSwC	M	NOV	3T	Refocusing after MW > Refocusing after mindful	4 A	↑L						
21	45	Taylor et al., 2011	Zen and Mindfulness	CSwC	EET	EX, NOV	3T	M vs. R in EX vs. NOV	2 A							
22	46	Tomasino and Fabbro, 2016	FAMM	CSwC	M and R (in blocks)	NOV	3T	Post-MT > Pre-MT	7 A	↑R		↑L			↑L	

Form of meditation: OM, open monitoring; FA, focused attention; LKM, Loving-Kindness; FAMM, focused attention-based mindfulness meditation. Study Design: WS, within-subjects; CS, cross-sectional; L, longitudinal, RCT, randomized controlled trial. Participants: EX, expert; NM, non-meditator; NOV, novice. Task/assignment: M, meditation; C, control task; R, rest; MM, mindfulness meditation; EET, emotional expectational task. Contrasts: MT, mindful training; MW, mind wandering; Mf, mindfulness; FB, focused breathing. Foci: A, activation; D, deactivations. Related brain regions: Brodmann area (BA) was followed when given, or designation provided by the authors. ND, no difference; R, right; L, left, B, bilateral; dACC, dorsal anterior cingulate cortex (BA 11, 24, 25, 32); DLPFC, dorsolateral prefrontal cortex (BA 9–12, 45, 46, 47); IPL, inferior parietal lobule (BA 39, 40, 43) (also called the Temporal–Parietal Junction or TPJ); AIC, anterior insular cortex (BA 13, 14, 47, 48); SPL, superior parietal lobule (BA 7, Precuneus); THAL, thalamus; BG, Basal Ganglia (Caudate, Putamen, Globus Pallidus).

### Data collection and extraction

Two reviewers independently searched the PubMed electronic database for relevant studies published up until October 2, 2021. The search strategy included a combination of keywords and MeSH terms related to Buddhist-derived Meditation Techniques (BdMT) on brain regions underlying tonic alertness or relaxation. Any discrepancies between the two reviewers were resolved through discussion or with a third reviewer.

After removing duplicates, the titles and abstracts of the remaining studies were screened for eligibility based on predetermined inclusion and exclusion criteria. Full texts of potentially relevant studies were then assessed for eligibility.

Data extraction was performed independently by two reviewers using a standardized data extraction form. Extracted data included study characteristics (e.g., author, year, country), participant characteristics (e.g., age, sex), intervention details (e.g., type of intervention, duration), outcome measures (e.g., primary and secondary outcomes), and results (e.g., effect sizes, statistical significance).

Any disagreements in data extraction were resolved through discussion or by consulting the third reviewer. If necessary, we contacted authors for additional information or data.

### Quality assessment

The risk of bias assessment for the included studies was conducted using the Cochrane Collaboration’s tool for assessing the risk of bias in randomized trials (Higgins et al., 2011). The following domains were evaluated:

(1) Random sequence generation: Was the allocation sequence adequately generated?
(2) Allocation concealment: Was allocation adequately concealed?
(3) Blinding of participants and personnel: Were participants and personnel blinded to the intervention?
(4) Blinding of outcome assessment: Were outcome assessors blinded to the intervention?
(5) Incomplete outcome data: Were incomplete outcome data adequately addressed?
(6) Selective reporting: Was the study free of selective outcome reporting?
(7) Other sources of bias: Were there other potential sources of bias, such as funding or conflicts of interest?

The risk of bias assessment (see Table 2) was performed independently. For each domain, we assessed the risk of bias as low, high, or unclear/not reported. Disagreements were resolved by consensus or consultation. The results of the risk of bias assessment are presented in Table 2.

**TABLE 2:** Results of the risk of bias assessment.

References	Random sequence generation	Allocation concealment	Blinding of participants and personnel	Blinding of outcome assessment	Incomplete outcome data	Selective reporting	Other sources of bias
Allen et al., 2012	Low	Low	Unclear	Unclear	Low	Low	Low
Baerentsen et al., 2010	High	High	Not reported	Unclear	Low	High	Low
Bauer et al., 2019	High	High	Not reported	High	Low	Low	Low
Brewer et al., 2011	Low	Low	Not reported	Low	Low	Low	Low
Dickenson et al., 2013	Low	Low	Not reported	Low	Low	Low	Low
Doll et al., 2016	Low	Low	Low	High	Low	Low	Low
Farb et al., 2007	Low	Low	Not reported	Unclear	Low	High	Low
Gard et al., 2012	High	High	Not reported	High	Low	Low	Low
Grant et al., 2011	Low	Low	Low	Unclear	Low	Low	Low
Hasenkamp et al., 2012	High	Low	Unclear	Unclear	Low	Low	Low
Hölzel et al., 2007	High	High	Unclear	High	Low	Low	Low
Ives-Deliperi et al., 2011	Low	Low	Unclear	Low	Low	Low	Low
Kim et al., 2020	Low	Low	Low	Low	Low	Low	Low
Klimecki et al., 2013	Low	Low	Unclear	Unclear	Low	Low	Low
Lee et al., 2012	Low	Low	Low	Unclear	Low	Low	Low
Lutz et al., 2015	High	High	Not reported	Unclear	Low	Low	Low
Manna et al., 2010	High	High	Not reported	Unclear	Low	Low	Low
Opialla et al., 2015	High	Low	Not reported	Unclear	Low	Low	Low
Pagnoni et al., 2008	Low	Low	Low	High	Low	Low	Low
Scheibner et al., 2017	High	High	Not reported	Unclear	Low	High	Low
Taylor et al., 2011	Low	High	Not reported	High	Low	Low	Low
Tomasino and Fabbro, 2016	Low	Low	Low	Low	Low	Low	Low

### Data analysis procedures

#### Loci/voxels selection

Over-all, 40 experiments, where all voxels that explicitly reflected significant arousal-related brain activity, were assessed. All the coordinate spaces (MNI vs. Talairach space and Talairach space vs. MNI) were made uniform by transforming coordinates reported in Talairach coordinates into the MNI space using the mni2tal algorithm (Lancaster et al., 2007) and vice-versa. The linear transformation was calculated using Yale BioImage Suite version 1.2.0, 2020/08/25.^1^ The current systematic review-cum-meta-analyses included data from 1,139 participants and 302 activation foci.

#### Statistical approach

A Statistical plot was developed using lists of *x*, *y*, and *z* coordinates in the MNI space. The meta-analysis was performed using the most recent version (Eickhoff et al., 2009, 2012) of the Ginger ALE 3.0.2 software^2^ for coordinate-based meta-analysis of neuroimaging results (Turkeltaub et al., 2002; Laird et al., 2005). An advantage of Ginger ALE is that it allows random-effects inference by evaluating for above-chance clustering of foci between experiments (Laird et al., 2005, 2009; Turkeltaub et al., 2012). Here, the factor of uncertainty, which is inherent to the actual location of the peaks, is accounted for by modeling each coordinate as a 3D Gaussian function with a 12-mm Full Width at Half the Maximum (FWHM) than a single point. Hence, the localization likelihood distributions define the chance that a provided focus truly lies within a precise voxel (Laird et al., 2005, 2009; Eickhoff et al., 2009, 2012). The statistical effectiveness (or significance) is achieved by a permutation test of randomly induced foci, employing the exact FWHM and the number of foci. Ginger ALE meta-analysis demonstrates that meaningful outcomes are achieved if the convergence throughout the meditation studies happens more plausible than anticipated. But this doesn’t mean that every single or even the bulk of the meditation studies activate a certain region (Eickhoff et al., 2009, 2012). The voxel-wise comparison is checked contrariwise to the null hypothesis of uniformly distributed peaks, presenting a group of ALE values critical for thresholding the probability map. We corrected for multiple comparisons (Laird et al., 2005, 2009; Eickhoff et al., 2009, 2012) using the uncorrected *p*-value at 1.0E−4 and false-discovery rate (FDR) at *p* < 0.05 and used a 0 mm^3^ volume to define a cluster. The ALE thresholded maps values were laid on a canonical T1-weighted structural scan make use of Mango, a multi-image analysis viewer.^3^ The ALE meta-analysis also provided other outputs, such as information of location, anatomical labels, and cluster size, among others.

## Results

After running the ALE meta-analysis, we didn’t find any significant activation with a false-discovery rate (FDR) at *p* < 0.05, when we used the uniformed MNI space as the input coordinates. But interestingly, we did find significant ALE activation when we inserted the uniformed Talairach space (see Supplementary Appendix Figure 2 and Supplementary Appendix Table 2). Further, we carried out the ALE meta-analysis with an uncorrected *P*-value of 1.0E−4 (using uniformed MNI space; see Supplementary Appendix Figure 1 and Supplementary Appendix Table 1) and we observed four brain regions - medial frontal gyrus (MFG), precuneus, insula, and inferior parietal lobule (IPL). In view of this review, we didn’t have any significant findings for uniform MNI space for FDR at *p* < 0.05, and as per the trend in neuroimaging studies, we decided to stick with using MNI space as the main reference for our results. To show credible and uniform anatomical terminology and labels across our meta-analytic study, we compared our coordinates of ALE activation with published meta-analyses, experimental data, and reviews. However, we did find significant results for the uncorrected *P*-value of 1.0E−4 using uniform MNI space. The same was confirmed when we used the uniform Talairach space coordinate system (see Supplementary Appendix Figure 3 and Supplementary Appendix Table 3).

### Medial frontal gyrus

Our ALE analysis displayed invariant activation within the medial frontal gyrus (MFG) in the right hemisphere (see Supplementary Appendix Table 2, Cluster 1). Coordinates in our ALE activation seem to correspond with posterior parts involving Brodmann areas 9, 10, and 32.

### Precuneus

The second consistent activation was revealed in the left Precuneus corresponding to the Brodmann area 31. And Brodmann areas 31 and 23 were only observed when ALE analysis was carried out with uncorrected *P*-value 1.0E−4 (see Supplementary Appendix Table 2, Cluster 2).

### Insula

The third activation observed in ALE analysis with uncorrected *p*-value only (see Supplementary Appendix Table 2, Cluster 3) was in the left insula corresponding with Brodmann areas 13.

### Inferior parietal lobule

The fourth activation revealed within the right inferior parietal lobule (IPL) was again only observed with uncorrected *p*-value (see Supplementary Appendix Table 2, Cluster 4) ALE analysis.

## Discussion

The current study seeks to come up with meta-analytical evidence of wake-promoting or arousal effects in Buddhist meditation by rendering from the recent neuroimaging works. In the Buddhist forms of meditation, mindfulness is one of the primary aspects of the technique, exercised by means of executive attention focused on the mind (thought), body, and speech (breathing). An analysis of the neuroimaging studies related to Buddhist meditation practices has been a complex and challenging work due to the following reasons: data collected from various studies comes from multiple forms of meditation (our study reports data collected from more than three types of Buddhist meditation); huge deviation in meditational experiences among different groups of practitioners, varying from a few weeks for novice participants going through MBSR training or comparable programs to hundreds and thousands of hours for adept practitioners and monks; examining various stages of Buddhist meditation (beginner, intermediate and/or advanced stages).

These disparities make it difficult to analyze and interpret the results in a consistent manner, which likely may lead to incorrect conclusions (Chiesa et al., 2011, 2013; Tang et al., 2012). Our systematic review-cum-meta-analyses study showed consistent activations in the right MFG and left precuneus despite above mentioned methodological concerns. Apart from these two invariant activations, the left insula and right IPL also contributed to ALE meta-analysis.

Wakefulness and sleep tendency are linked to distinctive brain regions, which are also noticed to get activated during different meditation practices - both during tasks/at rest (in a trait-related manner) and during meditation (in a state-related manner). In general, reduced alertness and the shift in the wakeful state to the sleeping state are linked to receding activity in most brain regions, mainly frontal regions, whereas the observed increase in activity in the default mode network (DMN) (Rolls et al., 2003; Picchioni et al., 2008; Olbrich et al., 2009). Tonic alertness is correlated with the activity of cortical regions and subcortical systems in the right hemisphere, especially the dorsolateral prefrontal cortex (DLPFC), the dorsal anterior cingulate cortex (dACC), the anterior insula (AC), the IPL, the thalamus, and the brain stem (Posner and Petersen, 1990; Sturm et al., 1999; Sturm and Willmes, 2001; Mottaghy et al., 2006; O’Connell et al., 2006, 2008; Sadaghiani et al., 2009, 2010; Langner and Eickhoff, 2013). Several fMRI investigations have observed that meditation-related activations are more concentrated in the right cerebrum, which very much corresponds to tonic alertness–related brain regions (e.g., inferior parietal lobule, lateral PFC, and anterior Insula) (Lazar et al., 2000, 2005; Farb et al., 2007; Lutz et al., 2008a; Zeidan et al., 2011; Hasenkamp et al., 2012; Sato et al., 2012).

Our systematic review-cum-meta-analyses show a string of activation clusters, located in two regions: right MFG, and left precuneus. Activation of MFG is considered to be engaged in both social cognition (Amodio and Frith, 2006; Molenberghs et al., 2016) and performance monitoring (Van Noordt and Segalowitz, 2012). The medial frontal gyrus (MFG) and precuneus are two brain regions that have been consistently implicated in the practice of Buddhist meditation. Several studies have reported that experienced meditators exhibit increased activation in these regions compared to novices during meditation. The precise mechanisms underlying the involvement of these regions in meditation are not yet fully understood, but several theories have been proposed (Tang and Tang, 2020). One theory suggests that the MFG is involved in attentional control and cognitive flexibility, which are key components of mindfulness meditation. This theory suggests that meditation may enhance these cognitive processes by strengthening the neural circuits in the MFG. Supporting this theory, a study by Tang et al. (2015) found that mindfulness meditation training was associated with increased activation in the MFG during a working memory task, suggesting that meditation may enhance attentional control and cognitive flexibility through its effects on this region. Another theory suggests that the precuneus is involved in self-referential processing and self-awareness, which are important aspects of meditation. This theory suggests that meditation may enhance self-awareness by strengthening the neural circuits in the precuneus. Supporting this theory, a study by Garrison et al. (2015) found that experienced meditators exhibited increased connectivity between the precuneus and other regions involved in self-referential processing compared to novices. Other studies have suggested that the MFG and precuneus may be involved in more general processes related to meditation, such as attention regulation and emotion regulation. For example, a study by Hölzel et al. (2011) found that mindfulness meditation was associated with increased activation in the MFG and precuneus during a task requiring emotion regulation. This finding suggests that these regions may play a role in regulating emotional responses during meditation. Overall, while the precise mechanisms underlying the involvement of the MFG and precuneus in meditation are not yet fully understood, several theories have been proposed based on their known functional significance in attentional control, self-awareness, and emotion regulation. Further research is needed to fully elucidate the specific mechanisms underlying the involvement of these regions in meditation and their potential applications in clinical and non-clinical settings.

Activation of MFG is accompanied by dorsolateral prefrontal cortex (DLPFC, BA 9), anterior prefrontal cortex (BA 10), and dorsal anterior cingulate cortex (dACC, BA 32). En masse, these front part areas are correlated with a broad spectrum of multifarious processes, which comprises language (Poldrack et al., 1999; Katzev and Tu, 2013), executive functions like attention (Japee et al., 2015), and capability to examine emotion and valence (Amodio and Frith, 2006). Also, it is a widely accepted view that the ACC has a specific function in observing and resolution of dissension by deciding within vying processing options based on limited prior internal conscious plans (Pardo et al., 1991), in orienting the body position in reaction to sensorial stimuli (Vogt, 2005), in executive functions (Pardo et al., 1991; Vogt, 2005) and in pain modulation (opioid activity and placebo agency) (Colloca and Benedetti, 2005; Price et al., 2008). Including many of its functions, the dACC is considered to regulate arousal through multiple thalamic nuclei and brain stem noradrenergic activations (Sturm and Willmes, 2001). The ACC has been involved in the volitional regulation of arousal. The left-sided ACC is suggested to be more related to decreased arousal and right-sided activation with increased arousal (Critchley et al., 2000, 2001). Buddhist meditation traditions are seen to be related to increased activity in the dACC, during both tasks (Farb et al., 2010; Grant et al., 2011; Zeidan et al., 2011; Allen et al., 2012; Gard et al., 2012; Lee et al., 2012) and meditation (Brefczynski-Lewis et al., 2007; Hölzel et al., 2007; Manna et al., 2010; Short et al., 2010; Hasenkamp et al., 2012). Further, DLPFC has been observed to have greater neural activity throughout different forms of Buddhist meditation (including focused attention) (Fox et al., 2016). Increased activity levels in the DLPFC correspond to alert wakefulness (Buchsbaum et al., 1989; Sawaya and Ingvar, 1989; Maquet et al., 1990), whereas reduced activity in DLPFC is linked to increased sleep propensity and fatigue (Buchsbaum et al., 1989; Maquet et al., 1996; Braun et al., 1997). Buddhist meditation traditions are seen to be related to increased activity in the DLPFC, both during tasks (Farb et al., 2007; Zeidan et al., 2011; Allen et al., 2012; Lee et al., 2012) and during meditation (Ritskes et al., 2003; Brefczynski-Lewis et al., 2007; Manna et al., 2010; Short et al., 2010; Yu et al., 2011; Hasenkamp et al., 2012).

The second activation cluster observed in our ALE analysis predominantly involves the left precuneus along with the engagement of the dorsal posterior cingulate cortex (dPCC, BA 31). Both precuneus and PCC are a part of the default mode network (DMN), along with the medial prefrontal cortex (mPFC) and inferior parietal lobule (IPL). And it has been demonstrated that DMN constitutes the neural basis of self-referential processing (for example mind-wandering) (Raichle et al., 2001; Greicius et al., 2003), and the areas associated with DMN are mainly accountable for the notion of self and self-representation (Northoff et al., 2006). Corroborating evidence indicates that meditation practices may be linked to decreased DMN activity (Farb et al., 2007, 2010; Travis et al., 2010; Brewer et al., 2011; Taylor et al., 2011; Zeidan et al., 2011; Berkovich-Ohana et al., 2012). Also, look into counter-examples (Goldin et al., 2009, 2012; Goldin and Gross, 2010), rather than indicating mental rousing or mind wandering, activity in DMN is related to reduced levels of alertness and attentiveness (Carciofo et al., 2014) and decreased sympathetic arousal (Nagai et al., 2004). The DMN encloses some of the only brain regions that boost their activity during the transition from wakefulness to sleep (stage 1) (Picchioni et al., 2008; Olbrich et al., 2009), and alertness can be pharmacologically induced by repressing the DMN (Hahn et al., 2007). Thus, mind wandering and DMN activation can be regarded as a cue of drowsiness and mental laxity on a continuum with dreaming and sleep (stage 1) (Fox et al., 2013). From the above stance, we can say that decreased activity in DMN/mind wandering is not a pacifying effect in regard to relaxation/sleep, but relatively a push in the contrasting direction i.e., toward increased vigilance and alertness that offsets mental laxness and sleepiness. Hence, any relevant alterations of the “self” (engagement of cognitive or bodily selves) following meditational experiences should epitomize within the DMN. It is observed that the deactivation of the PCC for expert meditators during meditation is less mind wandering (such as less association with cognitive self) or insignificant goal-achieving exercise as practicing meditation is something like finishing a “task” or assignment (Brewer et al., 2011). Furthermore, the substantial coupling between the DMN and relevant attention and cognitive control areas indicates that decreased mind wandering is possibly executed by means of improved attention control.

Both ALE results were obtained using FDR (only in Talariach space) and uncorrected *p-*value activated medial frontal gyrus and precuneus. The uncorrected *p*-value gives two additional clusters comprising left insula, and right IPL (see Supplementary Appendix). The left insula is involved in interoception, or the perception of bodily sensations, while the right IPL is associated with spatial attention and self-other processing. One theory suggests that during meditation, practitioners may engage in introspection and self-awareness, leading to enhanced interoception and self-other processing, which in turn may activate the left insula and right IPL. This idea is supported by a study that found greater activation in the left insula and right IPL during a focused-attention meditation task compared to a control task (Brefczynski-Lewis et al., 2007). Another possible explanation for the involvement of these regions in meditation is related to the concept of decentering, or the ability to observe one’s thoughts and emotions from a detached perspective. This ability has been linked to increased activity in the left insula and right IPL (Farb et al., 2013). Thus, it is possible that meditation training may enhance decentering abilities, leading to increased activation in these regions.

Additionally, the right IPL has been shown to be involved in attentional control and cognitive flexibility, and some studies have suggested that meditation may enhance these abilities (Tang and Posner, 2009; Tang and Tang, 2020). Thus, it is possible that increased activation in the right IPL during meditation reflects enhanced attentional control and cognitive flexibility.

In the neuroscience of meditation, Insula is observed to be a significant brain area. Invariably, it is noticed to sustain interoceptive awareness such as bodily internal and visceral states alongside meta-cognitive awareness (Fleming and Dolan, 2012; Fox et al., 2014; Tang et al., 2015). Apart from underlying interoception (Critchley et al., 2004), the anterior insula (AI) has also been suggested to be the central node of the intrinsic alertness network (Sadaghiani et al., 2010; Langner and Eickhoff, 2013), the substrate of basic consciousness or awareness (Craig, 2009), and corresponds with states of autonomic arousal and wakefulness during meditation (Brefczynski-Lewis et al., 2007; Lutz et al., 2008a). The insula has been seen to have greater activation both during tasks following mindfulness training (Farb et al., 2007, 2010; Grant et al., 2011; Taylor et al., 2011; Zeidan et al., 2011; Allen et al., 2012; Gard et al., 2012) and meditation (Manna et al., 2010; Hasenkamp et al., 2012). Further, insula (anterior part), ACC, and basal ganglia are actively involved in executive attention (or conflict monitoring and resolution) (Raz and Buhle, 2006; Petersen and Posner, 2012). A relatively recent meta-analysis of fMRI studies of meditation has revealed that the insula and ACC brain areas are vital for most meditation techniques and traditions (Fox et al., 2016; Raffone et al., 2019; Tang and Tang, 2020). Likewise, other cross-sectional studies conducted with expert meditators (having experience of thousands of hours), who practice one particular type of meditation technique (focused attention, FA) have exhibited that the insula and ACC display more significant activation corresponded to clinical sound-matched controls (Fox et al., 2016). Following the progress in alerting and executive attention, subsequent longitudinal fMRI investigations demonstrated that brief integrative body-mind training (IBMT) during resting state renders more significant regional blood flow for the insula and ACC in comparison with the relaxation training group (Tang and Posner, 2009; Tang et al. 2015; Lin et al., 2018; Tang and Tang, 2020). Results from our ALE analysis, together with the above findings, provide substantial and coherent evidence that the deployment of attentional control in meditation practice corresponds to the insula and ACC. Furthermore, Insula and ACC displayed enhanced activation after repeated trials, implying that they perhaps are the central agencies of behavioral improvement in attentional control, specifically for executive attention.

Therefore, the involvement of the left insula and right IPL in Buddhist meditation likely reflects the enhanced interoception, self-awareness, self-other processing, decentering abilities, attentional control, and cognitive flexibility that are often associated with meditation practice.

The findings on the involvement of the medial frontal gyrus (MFG), precuneus, insula, and inferior parietal lobule (IPL) in Buddhist meditation have implications for clinical practice. These brain regions are involved in various cognitive processes, including attention, emotional regulation, and self-awareness, which are relevant to many clinical disorders. For example, studies have suggested that dysregulation of the MFG and precuneus is associated with symptoms of depression, anxiety, and post-traumatic stress disorder (PTSD) (Koenigs and Grafman, 2009; Lanius et al., 2010). The involvement of these regions in meditation could therefore have implications for the treatment of these disorders. In addition, the insula and IPL have been implicated in the experience of chronic pain and body awareness, and meditation has been shown to have potential as a treatment for chronic pain (Zeidan et al., 2012). Thus, the findings suggest that meditation may have potential as a complementary therapy for a range of clinical disorders that involve dysregulation of the brain regions implicated in Buddhist meditation.

The increased activation of the MGF and precuneus during meditation has been associated with improvements in attention, emotional regulation, and self-awareness, which are important factors in the treatment of various psychiatric disorders such as anxiety, depression, and addiction. The insula and IPL, on the other hand, have been linked to interoception and empathy, which are critical for the treatment of somatic disorders and certain personality disorders. Furthermore, recent studies have also shown that mindfulness-based interventions, which involve the cultivation of attention and awareness through Buddhist meditation practices, can be effective in reducing symptoms of various psychiatric disorders and improving overall well-being (Brewer et al., 2011). Therefore, the current findings on the involvement of MGF, precuneus, insula, and IPL in Buddhist meditation may provide a neurobiological basis for the therapeutic effects of mindfulness-based interventions and could inform the development of new interventions for clinical populations (Van Dam et al., 2018).

The present study aimed to provide a systematic review and meta-analysis of neuroimaging studies that investigate the arousing effects of Buddhist meditation practices. However, the analysis of these studies has been complicated by several methodological concerns, including the use of various forms of meditation, differences in participants’ meditational experiences, and the examination of different stages of meditation. Therefore, the results should be interpreted with caution due to the inconsistency of methods used across the studies. Additionally, the sample size of the included studies was limited, which may affect the generalizability of the findings. Furthermore, the studies employed different measures to assess wake-promoting or arousal effects, which may limit the conclusions that can be drawn from the results. Moreover, the heterogeneity in participant characteristics, including age, gender, ethnicity, and cultural background, may have influenced the results and restricted the generalizability of the findings. One limitation of Activation Likelihood Estimation (ALE) in our meta-analysis is that it relies on reported coordinates from neuroimaging studies, which can introduce publication bias. Studies with statistically significant results are more likely to be published, leading to an overrepresentation of significant findings in the meta-analysis. This bias can affect the accuracy of the ALE results and may not fully represent the entire body of research on the given topic. Additionally, some of the included studies did not have control groups, which may limit the interpretation of the results. Thus, the current study’s findings should be interpreted cautiously, and future research should address these limitations to provide more robust evidence of the effects of Buddhist meditation practices on brain function.

## Conclusion

We performed the foremost meta-analysis on neuroimaging data related to arousing or wake-promoting effects in Buddhist meditation and determined five distinctive brain areas that were correlated to alertness or wakefulness during meditation. They were localized in the frontal and subcortical brain areas. Further, somewhat to our surprise, our ALE analysis failed to find evidence indicating the contribution of brain regions such as mPFC, superior parietal lobule, basal ganglia, and thalamus, which previous studies have suggested crucial to alertness-related Buddhist meditation (Manna et al., 2010; Raffone et al., 2019). This could be ascribable to a relatively smaller number of neuroimaging studies that utilized specific manipulations toward the effects of wakefulness in the different forms of Buddhist meditation. For future investigation, with a sufficient number of studies of different forms of meditation, it would be possible to carry out *post hoc* analyses of different subsets of meditation to tell apart the cortical activation linked with the different forms of meditation. Furthermore, we consider that future research will be able to link neural data to objective behavior by evaluating clinical data, which in turn may help the scientific community to further appreciate the mental health benefits of meditational practice focusing on alertness and wakefulness.

## Data availability statement

The original contributions presented in this study are included in this article/Supplementary material, further inquiries can be directed to the corresponding author.

## Author contributions

All authors listed have made a substantial, direct, and intellectual contribution to the work, and approved it for publication.

## Footnotes

1
https://bioimagesuiteweb.github.io/webapp/mni2tal.html

2
https://brainmap.org/

3
http://ric.uthscsa.edu/mango/
