# Supplementary material for: A systematic review and activation likelihood estimation meta-analysis of fMRI studies on arousing or wake-promoting effects in Buddhist meditation
Source: Front Psychol. 2023 Oct 27;14:1136983. doi: 10.3389/fpsyg.2023.1136983 (PMC10646186; doi:10.3389/fpsyg.2023.1136983)
Supplement: Supplementary file 1 [file Image_1.pdf]

Figure 1

Common activation area determined from the ALE-analysis of 22 fMRI studies on meditation: A. medial frontal gyrus (left) and precuneus (right), B. insula, C. inferior parietal lobule.

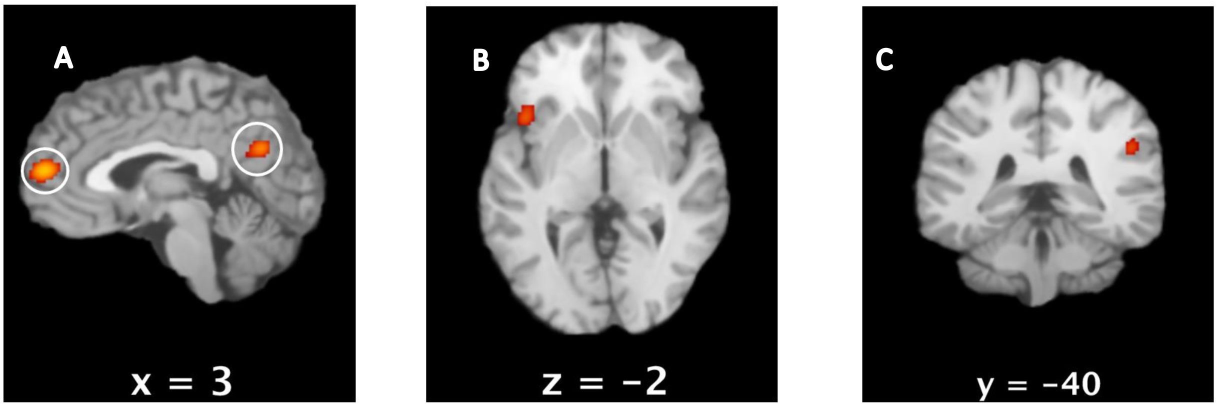

Note. Peak activation thresholded at uncorrected *p*-value of 1.0E-4, and coordinates stated in MNI space. X-, Y-, and Z-values correspond to sagittal, coronal, and axial planes respectively.
