# Supplementary material for: A systematic review and activation likelihood estimation meta-analysis of fMRI studies on arousing or wake-promoting effects in Buddhist meditation
Source: Front Psychol. 2023 Oct 27;14:1136983. doi: 10.3389/fpsyg.2023.1136983 (PMC10646186; doi:10.3389/fpsyg.2023.1136983)
Supplement: Supplementary file 2 [file Image_2.pdf]

Figure 2

Common activation area determined from the ALE-analysis of 22 fMRI studies on meditation. A. medial frontal gyrus (left circle) and precuneus (right circle), B. medial frontal gyrus, C. precuneus.

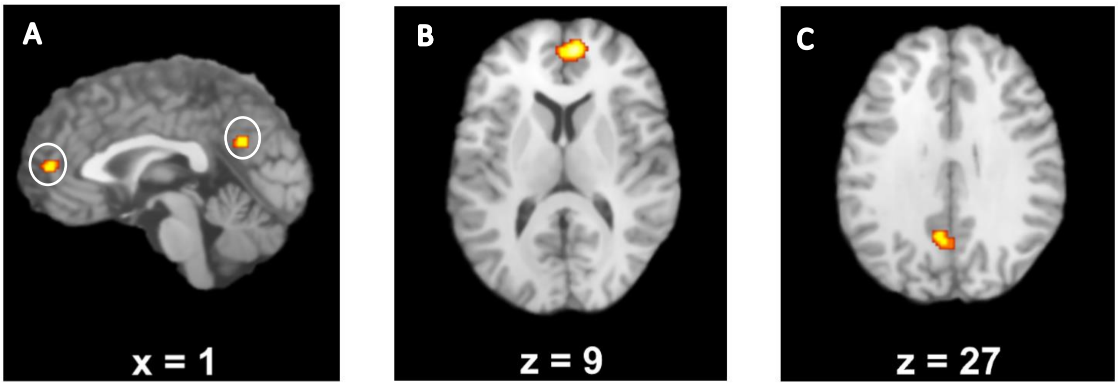

Note. Peak activation thresholded at false-discovery rate (FDR) at $p < 0.05$, and coordinates stated in the Talairach space. X- and Z-values correspond to sagittal planes and axial, respectively.
