# Supplementary material for: A systematic review and activation likelihood estimation meta-analysis of fMRI studies on arousing or wake-promoting effects in Buddhist meditation
Source: Front Psychol. 2023 Oct 27;14:1136983. doi: 10.3389/fpsyg.2023.1136983 (PMC10646186; doi:10.3389/fpsyg.2023.1136983)
Supplement: Supplementary file 3 [file Image_3.pdf]

Figure 3

Common activation area determined from the ALE-analysis of 22 fMRI studies on meditation: A. medial frontal gyrus (left circle) and precuneus (right circle), B. insula, C. inferior parietal lobule (right circle).

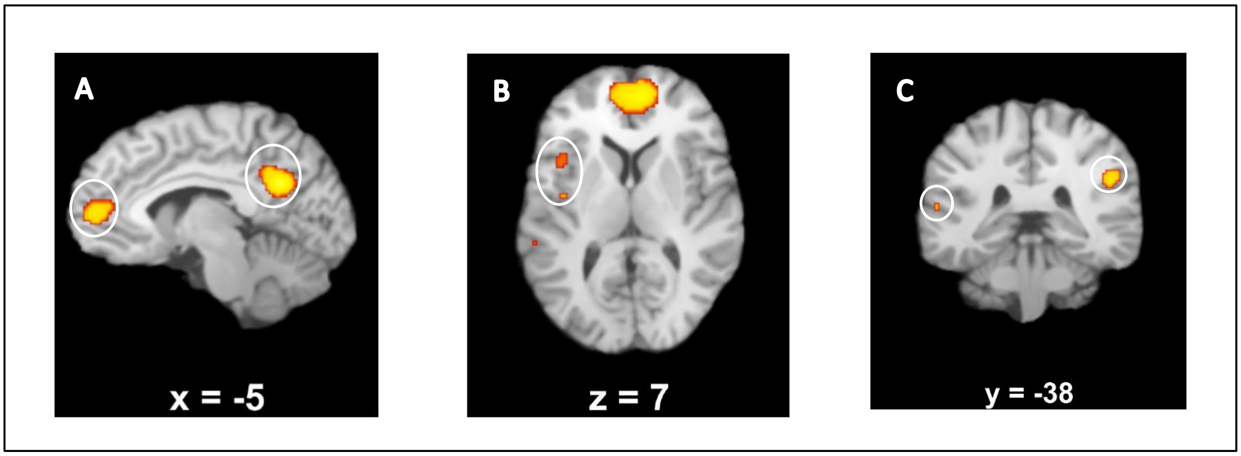

Note. Peak activation thresholded at uncorrected P value of 1.0E-4, and coordinates stated in Talairach space. X-, Y-, and Z-values correspond to sagittal, coronal, and axial planes respectively.
