# Supplementary material for: A systematic review and activation likelihood estimation meta-analysis of fMRI studies on arousing or wake-promoting effects in Buddhist meditation
Source: Front Psychol. 2023 Oct 27;14:1136983. doi: 10.3389/fpsyg.2023.1136983 (PMC10646186; doi:10.3389/fpsyg.2023.1136983)
Supplement: Supplementary file 4 [file Table_1.pdf]

**Table 1**

*Regions and clusters from the ALE meta-analysis using uncorrected p-value of 1.0E-4. Activation peaks are corrected above the threshold in MNI Coordinates with the maximum ALE-value depicted for each cluster. All activation peaks are allocated to the most plausible brain regions, as shown by the Mango multi-image viewer.*

| Cluster | Area | Brain Regions | Brodmann Area | MNI Coordinates | | | Cluster Size (mmˆ3) | ALE Max |
|---|---|---|---|---|---|---|---|---|
| | | | | X | Y | Z | | |
| 1 | RH | Medial Frontal Gyrus | 10, 9, 32 | 8 | 52 | 12 | 4816 | 0.0043 |
| 2 | LH | Precuneus | 31, 23 | -4 | -62 | 24 | 3104 | 0.004 |
| 3 | LH | Insula | 13 | -42 | 14 | -2 | 720 | 0.0037 |
| 4 | RH | Inferior Parietal Lobule | | 50 | -38 | 26 | 40 | 0.0036 |

*Note*. RH = right hemisphere; LH = left hemisphere
