# Supplementary material for: A systematic review and activation likelihood estimation meta-analysis of fMRI studies on arousing or wake-promoting effects in Buddhist meditation
Source: Front Psychol. 2023 Oct 27;14:1136983. doi: 10.3389/fpsyg.2023.1136983 (PMC10646186; doi:10.3389/fpsyg.2023.1136983)
Supplement: Supplementary file 6 [file Table_3.pdf]

Table 3

Regions and clusters from the ALE meta-analysis using uncorrected p-value of 1.0E-4. Activation peaks are corrected above the threshold in Talairach Coordinates with the maximum ALE-value depicted for each cluster. All activation peaks are allocated to the most plausible brain regions, as shown by the Mango multi-image viewer.

Cluster	Area	Brain Regions	Brodmann Area	Talairach Coordinates			Cluster Size (mmˆ3)	ALE Max
				X	Y	Z		
1	RH	Medial Frontal Gyrus	10, 9, 32	8	48	10	5752	0.0046
2	LH	Precuneus	31, 23, 7	-4	-60	24	4608	0.0045
3	LH	Insula	13	-40	10	2	1080	0.004
4	RH	Inferior Parietal Lobule		50	-36	26	568	0.0039

Note. RH = right hemisphere; LH = left hemisphere
